# Supplementary material for: A comparison of liver fat fraction measurement on MRI at 3T and 1.5T
Source: PLoS One. 2021 Jul 13;16(7):e0252928. doi: 10.1371/journal.pone.0252928 (PMC8277031; doi:10.1371/journal.pone.0252928)
Supplement: Attachment — Submitted filename: Response to Reviewers .docx [file pone.0252928.s001.docx]

**Response to Reviewers**

We thank the academic editor and reviewers for the comments which we hope have helped us improve the robustness and integrity of the manuscript. Thank you for giving us the opportunity to respond.

Academic Editor

1. Please ensure that your manuscript meets PLOS ONE's style requirements, including those for file naming.

*Done*

2.Please provide additional details regarding participant consent. In the ethics statement in the Methods and online submission information, please ensure that you have specified what type you obtained (for instance, written or verbal, and if verbal, how it was documented and witnessed). If your study included minors, state whether you obtained consent from parents or guardians. If the need for consent was waived by the ethics committee, please include this information.
Once you have amended this/these statement(s) in the Methods section of the manuscript, please add the same text to the “Ethics Statement” field of the submission form (via “Edit Submission”).

*Done*

3.Thank you for stating the following in the Competing Interests section:

"I have read the journal's policy and the authors of this manuscript have the following competing interests: Tim St Pierre holds shares in Resonance Health Ltd and consults to Resonance Health Ltd; Michael House holds shares in and is employed part time by Resonance Health Ltd; Wenjie Pang is employed by Resonance Health Ltd. Tim St Pierre and Michael House are inventors on a patent (No. 2012350165) for measuring liver fat. HepaFat-Scan® is owned and marketed by Resonance Health Ltd. The other authors have declared that no competing interests exist."

We note that one or more of the authors are employed by a commercial company: Resonance Health Ltd

a) Please provide an amended Funding Statement declaring this commercial affiliation, as well as a statement regarding the Role of Funders in your study. If the funding organization did not play a role in the study design, data collection and analysis, decision to publish, or preparation of the manuscript and only provided financial support in the form of authors' salaries and/or research materials, please review your statements relating to the author contributions, and ensure you have specifically and accurately indicated the role(s) that these authors had in your study. You can update author roles in the Author Contributions section of the online submission form.

Please also include the following statement within your amended Funding Statement.

“The funder provided support in the form of salaries for authors [insert relevant initials], but did not have any additional role in the study design, data collection and analysis, decision to publish, or preparation of the manuscript. The specific roles of these authors are articulated in the ‘author contributions’ section.”

If your commercial affiliation did play a role in your study, please state and explain this role within your updated Funding Statement.

b) Please also provide an updated Competing Interests Statement declaring this commercial affiliation along with any other relevant declarations relating to employment, consultancy, patents, products in development, or marketed products, etc.

Within your Competing Interests Statement, please confirm that this commercial affiliation does not alter your adherence to all PLOS ONE policies on sharing data and materials by including the following statement: "This does not alter our adherence to  PLOS ONE policies on sharing data and materials.” (as detailed online in our guide for authors [http://journals.plos.org/plosone/s/competing-interests](about:blank)). If this adherence statement is not accurate and there are restrictions on sharing of data and/or materials, please state these. Please note that we cannot proceed with consideration of your article until this information has been declared.

c) We note that you have a patent relating to material pertinent to this article. Please provide an amended statement of Competing Interests to declare this patent (with details including name and number), along with any other relevant declarations relating to employment, consultancy, patents, products in development or modified products etc. Please confirm that this does not alter your adherence to all PLOS ONE policies on sharing data and materials, as detailed online in our guide for authors [http://journals.plos.org/plosone/s/competing-interests](about:blank) by including the following statement: "This does not alter our adherence to  PLOS ONE policies on sharing data and materials.” If there are restrictions on sharing of data and/or materials, please state these. Please note that we cannot proceed with consideration of your article until this information has been declared.

Please include both an updated Funding Statement and Competing Interests Statement in your cover letter. We will change the online submission form on your behalf.

Please know it is PLOS ONE policy for corresponding authors to declare, on behalf of all authors, all potential competing interests for the purposes of transparency. PLOS defines a competing interest as anything that interferes with, or could reasonably be perceived as interfering with, the full and objective presentation, peer review, editorial decision-making, or publication of research or non-research articles submitted to one of the journals. Competing interests can be financial or non-financial, professional, or personal. Competing interests can arise in relationship to an organization or another person. Please follow this link to our website for more details on competing interests: [http://journals.plos.org/plosone/s/competing-interests](about:blank)

***Updated Funding Statement:***

*The study was supported by the National Institute for Health Research (NIHR) Leicester Clinical Research facility and patients were recruited through a Career Development Fellowship granted to GPM. The NIHR provided support in the form of salary for GPM, research materials and study consumables. The salaries for LA and GSG was supported by Clinical Research Training Fellowships from the British Heart Foundation (BHF). The funders did not have any additional role in the study design, data collection and analysis, decision to publish, or preparation of the manuscript.*

*Michael House is employed part time by Resonance Health Ltd; Wenjie Pang is employed by Resonance Health Ltd. This commercial affiliation did not influence the results of the study in any way. The specific roles of these authors are articulated in the ‘author contributions’ section.*

***Competing Interest Statement***

*Michael House holds shares in Resonance Health Ltd; Tim St Pierre and Michael House are inventors on a patent (No. 2012350165) for measuring liver fat. Tim St. Pierre was a former consultant for Resonance Health Ltd. HepaFat-Scan® is owned and marketed by Resonance Health Ltd. This does not alter our adherence to PLOS ONE policies on sharing data and materials*

Reviewer #1: 
1. The term volumetric liver fat fraction is misleading. This implies that you are measuring volumetric liver fat content, ie: fat content over the entire volume of the liver. I realize that this is not what is being measured, but I would strongly discourage the use of this term, at least without a strong qualification early in the paper, and should be removed from the title of the paper. The term VLFF is unfortunate and arises from the calibration and assumption that the authors have performed in previous work comparing their method to the steatosis area fraction. The underlying assumption that volume fraction and area fraction from a biopsy slide are equivalent is not well accepted, in my opinion. It is certainly true that those will correlate well with one another, but I think that the term volumetric fraction is unfortunate and misleading. This term should either not be used in this paper or a major qualifier that addresses my concern is needed in the introduction and the abstract. I would ask that this term not be included in the title of the paper.

*VLFF has been removed from the title and we have addressed the reviewers concerns regarding the 2-D nature of the HepaFat-Scan acquisition early in the paper on line 61. However, we disagree with the reviewer’s opinion (without evidence) that the assumption that the volume fraction and the area fraction in a thin section are numerically equivalent is not valid or well accepted. The Delesse Principle is a well-recognised principle relating areas of objects in thin section to their volume. The statement on line 56 has been reworded to be clearer and with specific references regarding the Delesse Principle.*

2. Related to the last point, a major limitation of this technique is that it is not “volumetric”. Rather, the method only measures liver fat over 3 slices. This is a major limitation for a variety of reasons including reproducibility for longitudinal studies. Unlike all commercial techniques that measure PDFF over the entire liver, ie: volumetric, the HepaFat-Scan technique does not. This is a problem for longitudinal reproducibility because it assumes that you can colocalize images across longitudinal studies, which is important because steatosis is known to be spatial heterogeneous. This limitation should be included in the Discussion. This is a same limitation that spectroscopy techniques have due to incomplete sampling of the liver (albeit not as bad for MRS).

*Thank you. This has now been added to the limitation section of the manuscript within the discussion.*

3. This paper would have been much stronger had it done a direct comparison with VLFF and PDFF. Given that PDFF only requires a single breath hold for the entire liver, and these methods are available on Siemens scanners, I am a little surprised that this wasn’t performed. If these data are available, I would highly recommend that you include them here. It would be good to understand the relative performance across field strength of the two techniques.

*Unfortunately PDFF measurements were not performed in these patients therefore this data is not available.*

4. Please specify that the HepaFat-Scan technique is a 2D technique. Please include the breath-hold time.

*Specified on line 61, breath hold time of 10 seconds also included now*.

5. In the Introduction, please avoid the term “global pandemic”. Bad choice of words in 2020.

*Duly noted and we apologise. This has been changed*.

6. In the last paragraph of the first page of the introduction describing a cohort of 25 obese individuals, this study was performed only with GE scanners, not Philips and GE. Related to this general area the authors are making a big deal out of the fact that there are no other studies comparing the reproducibility of 1.5T versus 3T for liver fat measurements with MRI. This is somewhat disingenuous. It is true that there are not many studies with direct comparison on the same day, in the same subjects. However, if you read the Yokoo meta-analysis paper in Radiology which had over 1,600 patients, you will note that a large number of these patients has both 1.5T and 3T using the same spectroscopy technique. Therefore, that paper was able to assess the reproducibility of PDFF measurements across field strength in a very large number of patients. Please include those numbers in the introduction and please tone down the language highlighting the fact that this study has such a large number of patients to assess reproducibility. I do agree that the current paper is the largest study (60 patients, 30 healthy controls) with direct same day comparison, but the way it’s currently phrased is a little misleading.

*We note and apologise for the error in referencing the study. The study referred to was Serai et al and this has now been corrected. Language in the paragraph has been changed and the Yokoo meta-analysis acknowledged. Table 2 in the Yokoo meta-analyses details that there are only 4 studies (Kang, Artz, Mashood, Serai) that measured PDFF at both 1.5 and 3T for the same cases and those four studies account for 80 of the 1960 cases in the Yokoo paper. These numbers have been acknowledged. Thank you.*

7. Not many technical details about the proprietary technique are provided. This is fine but is a weakness of the study and should be acknowledged in the discussion. A few other questions that are remaining: how is the spectral complexity of fat considered in the fit for water and fat signals, and how are phase shifts related to concomitant gradients in eddy currents handled? Is this a magnitude-based technique? A few more details to this effect would be reasonable and helpful to the reviewers.

*Thank you. The technical details of HepaFat-Scan are given within the manuscript and additionally within references 21 and 23. To further clarify, fat is modelled as single spectral component in this magnitude-based technique. As a magnitude-based technique the phase information is disregarded and hence the phase shifts related to eddy currents are not an issue. We have added additional detail on line 61.*

8. A limitation of the interstudy repeatability is that repeat scans 20 days after their baseline can lead to significant bias, especially if there is treatment that is occurring during this time (I believe this was one of the weight loss studies?). Further, variation in steatosis can occur over this time frame. The fact that 3T imaging occurred after 1.5T is another potential source of bias. These limitations should be addressed in the discussion.

*Thank you. These points have been added to the discussion section. 20 days was the upper limit in one patient, in most cases this was much lower and within 1-2 weeks, the scans were not performed on the same day as the patients came in fasted, had a clinical review and were already undergoing a 1.5T and 3T scan on that day, therefore were not willing or able to undergo a further 1.5T scan.*

9. First sentence of the discussion “largest single cohort of patients scanned on the same day across 2 field strengths”. Again, this is technically correct, but somewhat misleading considering the scope and magnitude of the Yokoo meta-analysis. Please see my comment above.

*We stand by this statement and the reviewer agrees it is correct. A meta-analysis is not a single cohort.*

10. The figures appear very pixelated in my version. I am assuming that this was related to the submission process. Assuming that they are higher resolution, the quality of the figures looks good.

*Figures have been edited and uploaded using PACE.*

11. The use of a single vendor for this comparison limits the generalizability of this study. In addition, the Siemens scanners are actually 2.89T, not 3.0T, thus in my opinion, this sort of study would also need to be performed on Philips and GE systems in order to demonstrate generalizability. A comment to this effect, including the fact that the Siemens magnet is lower than 3.0T, should be included in the Discussion.

*a) We agree and have always included this within the manuscript “but as this is a single centre study confined to two specific Siemens scanners, it cannot be implied the results are applicable to other models and manufacturers” within the limitations section.*

*b) We have consulted all the literature both from the manufacturer and previous papers on the subject. The Siemens Skyra is marketed, used for both research and clinically as a 3T MRI. All literature and publications on the subject use 3T, therefore we do not think it would be an accurate representation to imply it is a lower strength magnet.*

Reviewer #2: 
1. Introduction (Page 4, Row 53). At page 3 other common techniques are discussed for fat estimation using invasive a non-invasive techniques are discussed. However, page 4 starts with introducing VLFF without comparing it to the methodologies on page 3 more than it has comparable results to biopsy (based on ref 21). I miss a small motivation for VLFF as a technique. What benefits do it have compared to invasive methods or to spectroscopy and PDFF? Or are they totally interchangeable? Is there any situations where VLFF could be more beneficial?

*Compared to invasive methods, we allude to its’ benefits in “this is an invasive, uncomfortable procedure with significant procedural risks, including infection, and major haemorrhage. Liver biopsy is also subject to sampling variability. As such, appropriate non-invasive methods of liver fat measurements are desirable”. VLFF is presented as a comparable non-invasive alternative to PDFF*.

2. Method (Page 6, Row 107): The number of slices for the 3 T acquisition is missing.

*Same as 1.5T, specified now.*

3. Method (Page 7, Row 131): The algorithm addresses correction for confounding factors including correction for different T1 relaxations times between fat and water signal, T2*-correction etc. However, no remarks are adressessed regarding B0 and B1 inhomogeneity. Is the VLFF measurement sensitive to that and how would it affect the results?

*The use of magnitude data is expected to make the HepaFat-Scan technique insensitive to field inhomogeneities. The excellent agreement between HepaFat-Scan and biopsy results (Ref 21) would support this assessment.*

4. Method (Page 7, Row 151): Were there any reason for the “not on the same day” of the inter-scanner repeatability investigation at the 3T scanner? Would there be any benefits on testing the variability on the same day?

*There were various reasons for this, predominantly patient compliance and ease. As the visit included a clinical review and patients came in fasted, and already had a 1.5T and 3T scan on this day, they were not willing or able to have another cardiac MRI scan lasting 1.25 hours on the same visit*. *Another reason this was not done was to ensure informed consent for the repeatability study and clinical availability of the scanner.*

5. Results (Page 9, Row 186): Please include the confidence interval (CI) for the ICC calculations (both for 1.5 vs 3T comparison as well as the 3 T repeatability investigation. Rephrase (if needed) the statement of the agreement. Include the CI in Table 1 and abstract.

*Done*

6. Results (Page 9, Row 189) Table 1: Change “Values (CI)” change to “(95 % CI)”. Indicate the significance of the bias would help the interpretation of table 1 without needing to read the main text. Table 1 would increase the possibility to interpret the results if indicating the significant bias.

*Done*

7. Results (Page 9, Row 176; Page 25 (Figure2)) No Residual plot or any analysis is done to investigate a potential proportional bias of the correlation. An overall overestimation of 3T compared to 1.5 T is observed. Although the bias is small, it might be valuable to investigate any proportional biases if intended use is for diagnosing fatty liver. Especially since the cut-off between healthy and fatty liver is considered to be as low as 5 %. Is it therefore possible to include a report of the looking at the residual plot or add a regression line at Figure 2 (and 3)?

*We do not consider regression analysis as an appropriate approach to assess agreement between methods and regression is explicitly not recommended by Bland and Altman for method comparison studies. Inspection of the Bland-Altman plot in Figure 2 indicates that the standard deviation of the differences is approximately constant across the measurement range suggesting there is no significant proportional bias.*

8. Results (Page 25) Figure 2: Is it possible to increase the resolution of the Figure to increase readability? (In the PDF link I read and when I downloaded the .tiff image I found it hard to read figure 2 and figure 3. A higher resolution would increase the readability a lot.)

*Improved and uploaded using PACE*

9. Results (Page 26) Figure 3: Is it possible to increase the resolution of the Figure to increase readability? (In the PDF link I read and when I downloaded the .tiff image I found it hard to read figure 2 and figure 3. A higher resolution would increase the readability a lot. )

*Improved and uploaded using PACE*

10. Discussion (Page 11, Row 233): I find the paragraph starting with “It is also interesting to note that although the VLFF values between the two groups are very different…” is a bit out of scope, not necessarily correct, and hard for the reader to interpret themselves based on what is presented in the result section. The authors claim the VLFF clearly distinguish between health and diseased states but the only measurement we have access to as a reader is the mean value and standard deviation. So based on the population level, it seems that patients diagnosed with type II diabetes seems to have a higher fat fraction in there liver but we don't know the overlap or the distribution of the distribution of the fat content. However, the cohort are not included based on their known fatty liver status, but if they have a type II diabetes diagnose. I suggest removing this paragraph alternatively re-phrasing it to avoid misunderstandings. I don’t think the conclusions from that paragraph is possible based on the data that was provided.

*Noted and rephrased*

Minor issues
11. Results (Page 24) Figure 1: Is it possible to increase the size of number 1 and 2 of the ROI: s alternatively increase the resolution a bit for readability?

*Resolution has been improved and uploaded using PACE*

12. Results (Page 25 - Page 26) Figure 2 and Figure 3: In Figure 2, Bland-Altman is written with an “-“ in the middle. In Figure 3, Bland-Altman is written as to separate words. Consider being consequent.

*Figure 3 edited*

13. Results (Page 26) Figure 3: In figure 3, indicate the unit (%) on the axes.

*Done.*
